# Sleep Disturbances in Children With Autism Spectrum Disorder at a Malaysian Tertiary Hospital

**DOI:** 10.3389/fped.2020.608242

**Published:** 2021-01-18

**Authors:** Ho Wai Koo, Juriza Ismail, Wai Wai Yang, Syed Zulkifli Syed Zakaria

**Affiliations:** ^1^Department of Paediatrics, Hospital Tunku Azizah, Kuala Lumpur, Malaysia; ^2^Department of Paediatrics, Faculty of Medicine, National University of Malaysia, Kuala Lumpur, Malaysia

**Keywords:** autism spectrum disorder, children, sleep, sleep duration, sleep latency, sleep disturbances

## Abstract

**Introduction:** Children with autism spectrum disorder (ASD) have a variety of co-morbid medical problems, including sleep disturbances. Prevalence of sleep disorders has been reported to be higher in this group as compared to the general population. Identifying sleep problems in children with ASD may help increase awareness and improve the overall quality of care for them. The aim of this study was to determine the prevalence of sleep problems and associated factors in a group of Malaysian children aged 6–16 years, with ASD.

**Method:** This is a cross-sectional study at the Child Development Centre of UKM Medical Centre (UKM MC) on ASD children aged 6–16 years. Demographic data was obtained and the Sleep Disturbances Scale for Children (SDSC) questionnaire was completed by the main caregiver. Logistic regression analysis was used to determine factors related to higher total SDSC scores.

**Results:** A total of 128 patients were recruited (111 boys) with a median age of 8 years 3 months (IQR: 2 years 10 months). Forty-seven (36.7%) of them obtained total SDSC scores in the pathological range with 19 (14.8%) scoring high for overall disturbances and 28 (21.9%) for at least one subtype of sleep disorders: 25 (19.5%) DIMS, 18 (14.1%) SBD, 10 (7.8%) DOES, 5 (3.9%) DOA, 6 (4.7%) SWTD, and 3 (2.3%) SHY. More than half of the children (57.8%) were reported to have sufficient sleep duration of 8–11 h, but longer sleep latency of at least 15 min (82.8%). Half of the ASD children also had co-morbidities in which one-third (34.4%) had attention-deficit hyperactivity disorder (ADHD). Using logistic regression analysis, four factors were significantly associated with higher total SDSC scores; female gender (*p* = 0.016), older age group (11–16 years old) (*p* = 0.039), shorter sleep length (*p* = 0.043), and longer sleep latency (*p* < 0.001).

**Conclusion:** The prevalence of sleep disturbances is high among Malaysian children with ASD, especially DIMS. Female gender, older age group, shorter sleep length, and longer sleep latency were found to be associated with the sleep disturbances. Evaluation of sleep problems should form part of the comprehensive care of children with ASD.

## Introduction

Autism Spectrum Disorder (ASD) is a neurodevelopmental disorder characterized by persistent deficits in social communication and social interaction with restricted, repetitive patterns of behavior, interests, or activities (1). Children with ASD have a variety of co-existing medical problems such as learning difficulties, attention-deficit hyperactivity disorder (ADHD), feeding issues and sleep disturbances (2).

The presence of sleep disturbance may negatively influence the outcome in a child with ASD (3–5). Fadini et al. found a strong correlation between sleep disorders in children with ASD with behavioral problems such as social anxiety and depression, withdrawal, aggression and issues with attention and thought processing (3). Shreck et al. found that fewer hours of sleep per night predicted more prominent social skills deficits (4). These highlight the imperative for increased awareness and inclusion of managing sleep problems in children with ASD.

High prevalence of sleep problems among children with ASD had been regularly reported in developed countries. A population study in California by Krakowiak and colleagues found that 53% of children with ASD had at least one frequent sleep problem (6). Liu et al. conducted a parent-reported questionnaire study consisting of 167 children with ASD in Pennsylvania which found that 86% of these children had at least one sleep problem almost every day, including bedtime resistance, insomnia, parasomnias, sleep disordered breathing, morning rise problems, and daytime sleepiness (7). These sleep-related disorders may persist into adulthood among people living with autism. Øyane and Bjorvatn found a high prevalence of sleep problems in Norwegian adolescents and young adults with autism (aged 15–25 years) (8). A Canadian study concluded that these sleep disturbances are also commonly found in high-functioning adults with ASD (age range: 16–27 years); which was underreported but found through objective measures such as actigraphy and polysomnography (9).

A meta-analysis by Elrod and Hood revealed that children with ASD have shorter total sleep time, longer sleep latency, and lower sleep efficiency than typically developing peers (10). This meta-analysis involved 10 studies with a total of 343 children with ASD and 221 children with typical development. Several theories have tried to explain the possible etiology of sleep problems in these children. It is known that sleep-wake rhythms regulated by the neurohormone, melatonin; has a decreased nocturnal production in children with ASD (11–13) while alterations in the synchronization of their neurotransmitter systems such as serotonin (11) have also been implicated. There have been reported links identified between genes involved in melatonin synthesis and ASD which can explain the sleep disturbances observed in children with ASD (14).

Interestingly, a recent cross-sectional study conducted in Indonesia's Surabaya and Japan's Kobe showed contrasting level of prevalence for sleep problems in children with ASD as 60 and 16%, respectively (15). This suggests that there may be other factors that contribute to sleep problems in children with ASD apart from the disorder itself. As sleep habits and arrangements may differ across cultures and socio-economic environments, this may explain the differences in sleep disturbances as perceived by their caregivers. Many parents of children with ASD had reported that they themselves were not getting sufficient sleep time and this may be correlated with the children's sleep disturbances leading to higher stress levels in the parents (16–18). There is evidence that improving sleep quality in these children can result in positive outcomes for both the children's behavioral challenges and parental stress levels (19).

The aims of this study were to assess the prevalence of sleep disturbances among Malaysian children with ASD and factors associated with it.

## Methodology

Ethical approval was obtained from the UKM institutional research and ethics committee (Project code: FF-2018-079).

This cross-sectional study was carried out at the Child Development Centre (CDC) of Universiti Kebangsaan Malaysia Medical Centre (UKMMC) in Kuala Lumpur, Malaysia; from January 2018 to December 2018. Children diagnosed with ASD by a pediatrician in the CDC based on the Diagnostic and Statistical Manual of Mental Disorders, 5th Edition (DSM-5) (1) were identified during routine clinic visits. Those between 6 and 16 years old were recruited into the study if their parents were able to complete the questionnaires independently. Only one parent was recruited for each child. Children with comorbidities associated with sleep disturbance such as congenital malformations affecting the upper airway (e.g., Pierre-Robin syndrome), structural abnormalities of the central nervous system, known chromosomal abnormalities, or using medication(s) to promote sleep, were excluded.

Written informed consent for the study was obtained before a questionnaire booklet containing the Sleep Disturbances Scale for Children (SDSC) (20) and background information were given. Demographic details were collected, together with specific questions regarding co-morbidities that could affect sleep, use of electronic devices in the bedroom prior to sleep and the family's practice of co-sleeping. Co-sleeping was defined as the child sleeping in either the same room or the same bed with either a parent/both parents, a sibling/siblings, or other household members [e.g., grandparent(s)]. Patients' medical diagnoses, co-morbidities and medication use were obtained from their health records.

Confidentiality was maintained as each completed data collection sheet was individually numbered with no reference made to any identifying records. The data were subsequently analyzed anonymously.

### Questionnaire

#### Sleep Disturbances Scale for Children (SDSC) (20)

SDSC was first developed by Bruni and his colleagues in 1996 to assess a variety of behavioral patterns relating to children's sleep. It consists of 26 items rated on a Likert-scale [from 1 (never) to 5 (always)] on how often each item occurred in the last 6 months. The sum of scores yield a total sleep score (Total SDSC) and six sleep disturbance subtypes: difficulty in initiating and maintaining sleep/insomnia (DIMS), sleep breathing disorders (SBD), disorders of arousal/nightmares (DA), sleep-wake transition disorders (SWTD), disorders of excessive somnolence (DOES), and sleep hyperhidrosis (SHY). Higher T-scores are correlated with increased sleep disturbance. Standardized norms are available and raw scores are converted to T-scores, with a T-score of more than 70 (two standard deviations above the normative mean) regarded as pathological. The SDSC had been used in Malaysia previously for studies of children with cerebral palsy and epilepsy (21, 22). The questionnaire was translated into Malay and Mandarin Chinese respectively with adequate reliability and internal consistency reported (21). Therefore, all three languages were used in this study.

To best of our knowledge, this is the first study evaluating sleep disorder in ASD children in our country using SDSC.

### Statistical Analysis

A sample size of 125 was required to achieve a power of 0.8 and an alpha of 0.05. All data were coded and analyzed by using Statistical Package for Social Sciences (version 25; SPSS IBM Statistics, IBM Corp., USA). A *p* < 0.05 was considered to be statistically significant. Based on test for normality, the Kolmogorov-Smirnov statistic was 0.125 with a significance of *p* < 0.001; hence the SDSC scores collected were not normally distributed. Univariate analysis was performed using chi-squared test for categorical variables while Mann-Whitney *U* test was used to analyse continuous variables. Independent variables were entered in a single step using logistic regression analysis to determine factors associated with high Total SDSC scores.

## Results

There were 189 patients during the study period; four did not complete the questionnaire while 57 patients did not fulfill the inclusion criteria of age. Hence, 128 data sets were available for analysis. Based on the study population, the median age was 8 years 3 months (IQR: 2 years 10 months) with the youngest patient being 6 years old and the oldest being 16 years old.

Table 1 shows the characteristics of the children with ASD in the study population. There were 111 males and 17 females included in the study analysis. Most of the children in our study population were between the ages of 6–10 years old (*n* = 106, 82.8%) with the remaining aged 11–16 years (*n* = 22, 17.2%). Majority of the children with ASD were of Malay ethnicity (*n* = 81, 63.2%). This was followed by Chinese (*n* = 39, 30.4%), Indian (*n* = 6, 4.7%), and other ethnic groups (*n* = 2, 1.7%). This is consistent with the general racial composition of the local Malaysian community.

**Table 1:** Demographic characteristics of the children with ASD.

**Characteristics**	***N* = 128**
	***n* (%)**
**Gender**	
Males	111 (86.7%)
Females	17 (13.3%)
**Age Group (years)**	
6–10	106 (82.8%)
11–16	22 (17.2%)
**Ethnic Background**	
Malay	81 (63.2%)
Chinese	39 (30.4%)
Indian	6 (4.7%)
Others	2 (1.7%)
Co-Morbidities^*^	64 (50.0%)
ADHD	44 (34.4%)
Asthma	12 (9.4%)
Eczema	9 (7.0%)
Allergic rhinitis	6 (4.7%)
Epilepsy	6 (4.7%)
Others	6 (4.7%)

* *Each patient may have more than one medical co-morbid*.

Half of the patients included had a medical co-morbidity with ~1-3rd (*n* = 44, 34.4%) having attention-deficit, hyperactivity disorder (ADHD). A proportion of the children were atopic with 12 having bronchial asthma (9.4%), nine (7.0%) having eczema, and six (4.7%) having allergic rhinitis.

Table 2 shows the sleeping practices in our study population. Fifty-nine (46.1%) families reported using at least one electronic device in the index child's bedroom with a mean duration of usage daily of 0.55 h (*SD*: 0.80 h). The families predominantly practice co-sleeping as a common arrangement, *n* = 105 (82.0%), predominantly the child sleeps with the parent(s) only (*n* = 86, 67.2%). Other co-sleeping arrangements include sleeping with sibling(s) only (*n* = 20, 15.6%), parent(s) and siblings (*n* = 16, 12.5%), grandparents or domestic helper (*n* = 6, 4.7%).

**Table 2:** Sleep practices of children with ASD.

	***N* = 128**	**M (*SD*)**
	***n* (%)**	
**Presence of Electronic Devices in Child's Bedroom**	59 (46.1%)	
**Electronic Devices Used in Child's Bedroom**		
Television	20 (15.6%)	
Desktop computer	6 (4.7%)	
Laptop computer	7 (5.5%)	
Smartphone	47 (36.7%)	
Tablet	17 (13.2%)	
**Length of time of Electric Devices Used in Child's Bedroom (hours/day)**		0.55 (0.80)
**Presence of Co-Sleeping**	105 (82.0%)	
**Co-Sleeping Partners**		
Parent (s)	86 (67.2%)	
Sibling(s)	20 (15.6%)	
Parent(s) AND sibling (s)	16 (12.5%)	
Others (e.g., grandparents/domestic helper)	6 (4.7%)	

Table 3 shows the sleep in the children with ASD. Although majority of these children (*n* = 74, 57.8%) had a normal duration of sleep (8–11 h), the remaining 54 (42.2%) had insufficient sleep duration (<8 h). Most of the children with ASD (*n* = 106, 82.8%) had longer sleep latencies (between 15 and 30 min).

**Table 3:** Sleep characteristics in children with ASD.

	***N* = 128**
	***n* (%)**
**Sleep duration (hours)**	
8–11 h	74 (57.8%)
5– <8 h	52 (40.6%)
<5 h	2 (1.6%)
**Sleep latency (hours)**	
<15 min	22 (17.2%)
15– <30 min	60 (46.9%)
>30 min	46 (35.9%)

Table 4 shows the SDSC scores in the children with ASD. Forty-seven children (36.7%) had at least one sleep disturbance with a subscale score or total SDSC score above the normative mean. Nineteen children had a pathological total SDSC score (14.8%). Difficulty in initiating and maintaining sleep (DIMS) had the highest prevalence; *n* = 25 (19.5%) followed by sleep breathing disorders (SBD); *n* = 18 (14.1%) and disorders of excessive somnolence (DOES); *n* = 10 (7.8%).

**Table 4:** Prevalence of sleep disturbances among children with ASD.

**Sleep parameters**	***N* = 128**
	***n* (%)**
At least one subscore or total score	47 (36.7%)
**Subtypes**, ***n*** **(%)**	
Difficulty in initiating and maintaining sleep	25 (19.5%)
Sleep breathing disorders	18 (14.1%)
Disorders of arousal	5 (3.9%)
Sleep-wake transition disorders	6 (4.7%)
Disorders of excessive somnolence	10 (7.8%)
Sleep hyperhidrosis	3 (2.3%)
Total SDSC Score	19 (14.8%)

*Presence of sleep disturbances when T-score >70 which is >2SD above the normative mean and treated as pathological*.

Table 5 shows the factors associated with pathological SDSC scores. Girls are significantly more predisposed than boys to a pathological score for at least one subtype or total SDSC score. Other factors such as presence of other medical co-morbidities such as ADHD, co-sleeping and duration of usage of electronic device in the bedroom were not associated with higher scores for each sleep disturbance category or the total SDSC score.

**Table 5:** Factors associated with pathological SDSC scores.

	**At least one subtype, *n***	**Total SDSC, *n***
**Gender**		
-Boys	38	13
-Girls	9	6
	(*p* = 0.037^*^)	(*p* = 0.043^*^)
**Co-morbid**		
-ADHD	19	10
-Asthma	7	4
-Allergic rhinitis	4	2
-Eczema	3	2
	(*p* = 0.237)	(*p* = 0.418)
**Co-sleeping**		
-Yes	38	16
-No	9	3
	(*p* = 0.590)	(*p* = 0.585)
	**Subtype score**	**Total SDSC score**
Electronic device use in the bedroom	*r*_s_ = 0.237	*r*_s_ = 0.061
	*p* = 0.312	*p* = 0.497

* *p < 0.05 taken as significant*.

Logistic regression analysis was performed to explore the effect of sleep length and latency, social, medical and co-sleeping factors on total SDSC scores; as depicted in Table 6. Input data for the regression model are as described: “Shorter sleep length” is defined as sleep length <8 h; “longer sleep latency” is defined as sleep latency of more than 15 min, “older age group” includes children 11–16 years old and gender comparison between males and females. The output data assesses the odds ratio of a child with ASD with a higher total SDSC score (pathological T-score of >70). Four factors were significantly associated with higher total SDSC scores; female gender (*p* = 0.016), older age group (11–16 years old) (*p* = 0.039), shorter sleep length (*p* = 0.043), and longer sleep latency (*p* < 0.001).

**Table 6:** Logistic regression analysis of factor associated with pathological total SDSC scores in children with autism spectrum disorder.

**Factor**	**B-Coefficient (SE)**	**Wald**	**Degree of freedom**	***p*-value**	**Odds ratio**	**95% confidence interval**
**Shorter sleep length**	3.411 (0.042)	5.719	1	**0.043**	3.49	**2.318 – 7.564**
**Longer sleep latency**	5.242 (0.648)	1.619	1	**<0.001**	1.33	**3.674 – 8.045**
**Older age group**	5.576 (0.267)	4.143	1	**0.039**	1.89	**0.3900 – 11.646**
**Female gender**	4.247 (2.129)	1.514	1	**0.016**	1.75	**0.549 – 7.450**
Longer duration of electronic device use prior to sleep (hours)	0.825 (0.054)	1.138	1	0.172	1.43	0.537 – 2.179
Co-sleeping	6.937 (3.408)	0.221	1	0.591	1.37	3.231 – 9.312
Comorbid of ADHD	1.495 (0.103)	2.207	1	0.141	1.73	0.740 – 5.154

*Bold value indicates significant p-value <0.05*.

## Discussion

Our study showed 36.7% of Malaysian children with ASD had indicators for at least one type of sleep disturbance which was comparable with other studies which estimated between 16 and 86% (3, 4, 7, 15, 23, 24) This value was higher compared to a previously reported 13.5% among typically developing Malaysian school-going children (25).

Nineteen children with ASD (14.8%) had a pathological total SDSC score which reflects an overall problem with sleep despite not having any specific subtype of sleep disturbance. Generalized disturbance in sleep may be attributed to a multitude of internal and external factors (3). Higher prevalence of these problems can be explained by the different circadian cycle in relation to the neurotransmitters such as melatonin, GABA and serotonin; which in turn can affect sleep cycles in children with ASD (11–14).

All the six subtypes of sleep disorders were present in different percentages in our children with ASD. The indicators for DIMS was the most prevalent. Approximately one-fifth of our patients had high DIMS scores suggesting that they have problems falling asleep and getting sufficient sleep. These data are in agreement with the values reported in the literature (15, 26, 27).

Slightly more than half of the children with ASD were reported to have a normal sleep duration (8–11 h) with the remainder 42.2% appearing to have shorter sleep duration which can translate to behavioral problems. A study conducted in England found that children with ASD slept for 17–43 min less each day than contemporary controls whereby the reduction was due to later bedtimes and earlier waking times (28). Other studies also supported that children with ASD have shorter total sleep time compared to typically developing peers (10, 28). This is congruent to other reports range of duration (8–10 h) (28–30). We found that more than 2/3 of the children with ASD in our study had prolonged sleep latencies with 1/3 exceeding 30 min. One possible reason for this may be the innate characteristic of sensory over-responsiveness in children with ASD which present a challenge for caregivers to settle the child to sleep at bedtimes (29). This warrant further investigation as it is a modifiable factor.

We found that the female gender is associated with a higher total SDSC score. This finding was not stated in previous studies on sleep disturbances in children with ASD and typically-developing children. It has been postulated that females with ASD have more dysregulation in their neurotransmitter system compared to their male counterparts (3, 24, 31).

Our study found that the older age group (11–16 years old) was significantly related to a higher total SDSC score. This is supported by previous studies involving typically-developing children as well as children with ASD (23, 24). This is most likely due to greater autonomy in older children and adolescents with regards to their bedtime. They are also more likely to have their own electronic devices. In addition, most of the children in these age group sleep separately from their parents.

Our study did not find a significant association between having a medical co-morbidity with more sleep disturbances as compared to some studies (3, 23, 27, 28). Some of our patients may have undiagnosed co-morbidity or the severity of their symptoms have been underreported.

Co-sleeping was a common practice in our study population, but this was not associated with more reported sleep disturbances. This contrasted with an earlier report that a relationship exists between co-sleeping and more sleep problems in children with ASD (15). Socio-cultural practices and differences in home arrangements may account for this, as earlier studies on Malaysian children with epilepsy and cerebral palsy revealed a high rate of co-sleeping but did not find a correlation with sleep disturbances (21, 22).

### Limitations

There were several limitations in this study. There was no comparative data with typically-developing children from the general population. In addition, our study population consisted of patients from a tertiary urban hospital; hence, the results may not be generalizable to the community.

SDSC is a screening tool for sleep disturbance originally developed for children from the general population. No specific questionnaire has been developed to ascertain sleep disturbance in ASD children. Efforts to create a questionnaire assessing sleep for children with ASD should be pursued. Questionnaire-based studies are subject to reporter bias and recall bias. Therefore, sleep diaries and polysomnography studies are objective measures which could help verify some of the reported sleep disturbances in the child.

## Conclusion

Sleep disturbances are common in Malaysian children with ASD, the most frequent indicators include disorders of initiating and maintaining sleep (DIMS), sleep breathing disorders (SBD) and disorders of excessive somnolence (DOES). We found an association between female gender, older age group, shorter sleep length, and longer sleep latency with the child's sleep problems. Clinicians who work with children with ASD should include evaluation of sleep problems as part of their comprehensive care. Affected children could then be directed to appropriate avenues for intervention.

## Data Availability Statement

The original contributions presented in the study are included in the article/supplementary material, further inquiries can be directed to the corresponding author/s.

## Ethics Statement

Ethical approval was obtained from the Universiti Kebangsaan Malaysia institutional research and ethics committee (Project code: FF2018-079). Written informed consent for the study was obtained from the main caregivers.

## Author Contributions

HWK, WWY, and JI contributed to the concept, design of the study, and drafted the manuscript. HWK and SS organized the database and performed the statistical analysis. All authors contributed to manuscript revision, read, and approved the submitted version.

## Conflict of Interest

The authors declare that the research was conducted in the absence of any commercial or financial relationships that could be construed as a potential conflict of interest.

## Abbreviations

DA: disorders of arousal
DIMS: disorders of initiating and maintaining sleep
DOES: disorders of excessive somnolence
SBD: sleep breathing disorders
SDSC: Sleep Disturbance Scale for Children
SHY: sleep hyperhidrosis
SWTD: sleep-wake transition disorders.
